# Regulation of immunity during visceral *Leishmania* infection

**DOI:** 10.1186/s13071-016-1412-x

**Published:** 2016-03-01

**Authors:** Vasco Rodrigues, Anabela Cordeiro-da-Silva, Mireille Laforge, Ricardo Silvestre, Jérôme Estaquier

**Affiliations:** CNRS FR3636, Université Paris-Descartes, Paris, France; Instituto de Investigação e Inovação em Saúde, Universidade do Porto, Porto, Portugal; Departamento de Ciências Biológicas, Faculdade de Farmácia, Universidade do Porto, Porto, Portugal; School of Health Sciences, Life and Health Sciences Research Institute (ICVS), University of Minho, Braga, Portugal; ICVS/3B’s-PT Government Associate Laboratory, Braga, Guimarães Portugal; Centre de Recherche en Infectiologie, Université Laval, Québec, Canada

## Abstract

Unicellular eukaryotes of the genus *Leishmania* are collectively responsible for a heterogeneous group of diseases known as leishmaniasis. The visceral form of leishmaniasis, caused by *L. donovani* or *L. infantum*, is a devastating condition, claiming 20,000 to 40,000 lives annually, with particular incidence in some of the poorest regions of the world. Immunity to *Leishmania* depends on the development of protective type I immune responses capable of activating infected phagocytes to kill intracellular amastigotes. However, despite the induction of protective responses, disease progresses due to a multitude of factors that impede an optimal response. These include the action of suppressive cytokines, exhaustion of specific T cells, loss of lymphoid tissue architecture and a defective humoral response. We will review how these responses are orchestrated during the course of infection, including both early and chronic stages, focusing on the spleen and the liver, which are the main target organs of visceral *Leishmania* in the host. A comprehensive understanding of the immune events that occur during visceral *Leishmania* infection is crucial for the implementation of immunotherapeutic approaches that complement the current anti-*Leishmania* chemotherapy and the development of effective vaccines to prevent disease.

## Background

*Leishmania* is a genus of kinetoplastid eukaryotes whose life-cycle relies on continuous shuttling between a mammalian host and an insect vector. These protozoans have a digenetic lifestyle, undergoing dramatic morphological changes to ensure adaptation and survival in either host. Within the gut of the sandfly vector, *Leishmania* endures as extracellular, flagellated and motile promastigotes. Conversely, in mammalian hosts, parasites survive and replicate inside host’s phagocytes as non-motile, round and obligate intracellular amastigotes. The continuous replication of amastigotes inside macrophages leads to apoptotic cell death of the host cell. The ingestion of apoptotic bodies and membrane blebs containing viable parasites by neighboring phagocytes allows a silent propagation of the infection [1].

More than 30 species of *Leishmania* have been identified, of which about 20 are human pathogens. The leishmaniases are divided into three medical conditions that involve cutaneous, mucocutaneous or visceral pathology. Virtually, all the mortality associated with the leishmaniases results from visceral disease. Due to the remote location of many visceral leishmaniasis (VL) endemic areas, the degree of under-reporting is severe. Conservative assumptions estimate the annual incidence at 400,000 cases, with about 20,000 to 40,000 associated deaths [2]. The primary VL endemic foci are located in the Indian sub-continent and East Africa, accounting for approximately 90 % of all cases [2]. *L. donovani* causes VL in Asia and East Africa, while *L. infantum* accounts for visceral disease in the Mediterranean basin and South America [3].

Visceral leishmaniasis has an asymptomatic incubation period of variable duration and early symptoms include intermittent fever, malaise and shivering. Overt disease manifests by striking splenomegaly, accompanied or not by hepatomegaly. In cases of concurrent VL and Acquired Immunodeficiency Syndrome (AIDS), splenomegaly may be absent. The hyperplasia of the reticuloendothelial system is accompanied by wasting and pallor of the mucous membranes [3–5]. Mononuclear phagocytes in the spleen, liver, bone marrow and lymph nodes appear heavily parasitized, but lymphocyte infiltration is usually scarce. In the spleen, atrophy of the white pulp is common, with loss of the architectural organization of lymphoid structures. Plasma cells are abundant in the spleen, and probably associated with the occurrence of polyclonal hypergammaglobulinemia. Anemia, thrombocytopenia and neutropenia are frequent and reflect both splenic sequestration and suppression of bone marrow function. Immune complexes are present, sometimes associated with nephritis, proteinuria and microscopic hematuria [3–6]. At advanced stages, thrombocytopenia along with prothrombin depletion leads to severe mucosal hemorrhage. Jaundice and ascites also occur at advanced disease. Secondary infections, particularly tuberculosis and pneumonia, become common and are frequent causes of death [5].

Immunity to *Leishmania* has long been known to depend on the development of type I immune responses characterized by initial production of Interleukin-12(IL-12) by antigen-presenting cells (APCs) that induce Interferon-γ(IFN-γ)-secreting Th1 T cells [7]. These, in turn, will induce the activation of the macrophage’s microbicidal mechanisms; in particular they induce the production of nitric oxide (NO) and reactive oxygen species (ROS), which are highly effective in killing intracellular amastigotes [8]. However, early studies noticed that VL progresses even in the presence of detectable levels of T helper-1 (Th1) cytokines, whose action is neutralized by immunosuppressive factors, such as IL-10 [9–11].

Here, we will review the immune events occurring in visceral organs, focusing on the spleen and the liver, during the acute and chronic stages of VL. By highlighting the main immune parameters associated with parasite persistence vs. parasite elimination, we aim to provide a concise picture of the immunology of VL that may help in the development of new therapeutic strategies.

## Review

### Studying the immunology of visceral leishmaniasis: animal models and human patients

Due to the intrusive procedures required to study infected organs in VL patients, the bulk of the knowledge concerning the regulation of immunity during VL has been obtained from mouse models [12]. In murine VL, the terms resistance and susceptibility refer to the ability of the host to rapidly control parasite growth. Indeed, susceptible strains such as the Balb/c develop a life-long chronic infection, which unlike humans is not fatal to the host. Most studies in mice are based on intravenous or intraperitoneal injection of a high dose of parasites, hence bypassing the early events on the skin and parasite navigation to the viscera [13]. Some comparative studies suggest that the parasite dose and inoculation route influence the kinetics of parasite colonization of the viscera and the ensuing immune response [14, 15]. Acknowledging these limitations, researchers are starting to employ alternative animal models of the disease to perform in-depth immunologic studies, such as the extremely susceptible Syrian hamster model, through sand-fly-mediated parasite inoculation [16]. Also, the recent use of rhesus macaques as models of VL takes advantage of the close phylogeny between humans and non-human primates and provides a window to the early events after infection which are silent in humans and hence not accessible [17, 18].

In mice, VL provides a clear example of organ-restricted immunity. In the liver, infection is self-resolving, in a manner that is dependent on the development of T cell-mediated immunity and formation of granulomas [19]. In contrast, in the spleen the immune system fails to clear parasites and instead, a lifelong chronic infection persists associated with immunopathology [20]. The compartmentalized immune responses clearly observed in murine VL are not evident in human patients, where infection is progressive and varying degrees of parasite load are observed in the viscera [21]. Also, in the Syrian hamster, parasites grow unimpaired in the spleen, liver and bone marrow (BM), until animal demise [22]. Finally, in our recent study in *L. infantum*-infected rhesus macaques, we observed a progressive increase in the parasite load in visceral organs as the infection advanced toward the chronic phase (8 months) [17]. Nevertheless, the compartmentalized immune response observed in mouse VL has been instrumental in defining the immune networks that dictate parasite elimination vs persistence during visceral *Leishmania* infection. We will address these events in the following sections.

### Mechanisms underlying the control of hepatic infection in mice

Liver resident Kupffer macrophages harbor most parasites after intravenous injection of mice with *L. donovani* or *L. infantum* [23, 24]. Kupffer cells have reduced innate capacity to kill intracellular *Leishmania* and hepatic parasite burden increases rapidly during the first weeks [25, 26]. Restriction of liver parasite numbers parallels the assembly of inflammatory structures, known as granulomas, constituted by a central core of fused and parasitized Kupffer cells and an outer cuff of motile lymphocytes and variable amounts of other immune cells [24, 27, 28]. Granulomas allow the local concentration of inflammatory cytokines that in turn efficiently activate the leishmanicidal mechanisms of Kupffer cells [29]. The kinetics of granuloma maturation during experimental infection of mice with *L. donovani* has been dissected in detail [27, 29, 30]. Interestingly, Kupffer cells exposed to the inflammatory environment during infection, but not directly infected by the parasite, appear activated a few hours after parasite inoculation and play a crucial role in initiating the protective response [31], by secreting several chemokines and cytokines that recruit immune populations, including monocytes, neutrophils and invariant natural T killer (iNKT) cells [32–34]. INKT cells, in particular, play a major role in coordinating initial granuloma formation [35]. Via their invariant T-cell receptor (TCR), iNKT cells recognize CD1d-bound lipophosphoglycan (LPG), the most abundant surface glycolipid of *Leishmania* spp., triggering early production of IFN-γ [36]. Additionally, iNKT cells rapidly secrete several cytokines upon activation, including C-X-C motive chemokine-10 (CXCL10), which attracts T cells and promotes maturation of granulomas [34, 35, 37]. However, the role of iNKT cells during VL is unclear, as their activation with selective ligands was contradictorily associated to disease amelioration or aggravation in independent studies [38, 39]. It is possible that these cells are important in the orchestration of the initial response after infection, but their chronic activation is detrimental to the host [39].

By one week after infection, T cells are recruited to the granuloma and eventually become the predominant immune cell type [40]. Given the low level of innate parasite killing in the early stages of infection in the liver, it has long been assumed that the majority of parasite-specific T cells were primed in the spleen and subsequently migrated to the liver, guided by chemotactic gradients [30]. Nevertheless, a recent study demonstrated that specific CD4 T cells can be primed in the liver and suffice to confer hepatic immunity [41]. Both CD4 and CD8 T cells appear indispensable for the development of mature granulomas [40]. Two-photon imaging revealed that antigen presentation to CD8 T cells is restricted to Kupffer macrophages [42], whereas CD4 T cells may be activated by both Kupffer cells and some granuloma-associated dendritic cells (DCs) [13, 29].

A number of cytokines play critical roles in granuloma development and parasite killing. IL-12 is produced by activated Kupffer macrophages and induces IFN-γ by granuloma-associated lymphoid cells [43]. In turn, IFN-γ maximizes the leishmanicidal capacity of Kupffer cells [13]. Arguably, the most important soluble factor for granuloma development and hepatic control of *Leishmania* infection is Tumor Necrosis Factor (TNF), which plays a crucial role in coordinating the assembly and maturation of granulomas [20]. In the absence of *TNF*, parasite growth in the liver proceeds unimpaired during the first weeks due to completely absent granuloma formation. However, later in infection (6–8 weeks) there is an abrupt assembly of granulomas causing rapid death due to fulminant hepatic necrosis [44, 45]. Additionally, lymphotoxin-α, a TNF-related cytokine, promotes the recruitment of leukocytes from the perivascular space to the sinusoidal areas, where infected Kupffer cells reside [46].

Granulomas attain full maturation by 2–4 weeks after infection and hepatic parasite burden rapidly declines up to 8 weeks post-infection [27]. Importantly, sterile immunity in the liver is not achieved. However, the presence of a residual parasite population is thought to incite a small but enduring immune response that provides long-term immunity to reinfection [13].

### Early events in the spleen during visceral *Leishmania* infection

The spleen is the body’s largest blood filter. Splenic macrophages are strategically placed to remove any exogenous particle or pathogen that enters the spleen through the blood stream [47]. Following intravenous injection of *L. donovani*, about 95 % of the parasites are phagocytized by three distinct splenic populations; red pulp macrophages, marginal zone macrophages (MZM) and marginal metallophilic macrophages (MMM) [48]. Unlike liver Kupffer cells, macrophage populations of the spleen demonstrate a remarkable innate capacity to kill the parasite. Indeed, it is estimated that 50 % of the initial parasite inoculum is killed by macrophage populations of the marginal zone within the first 24 hours after infection [48]. For both MZMs and MMMs this was shown to depend on the recruitment of the Interferon Regulatory Factor (IRF)-7 to parasite-containing phagosomes and may involve leishmanicidal mechanisms independent of NO [49].

**Fig. 1:**
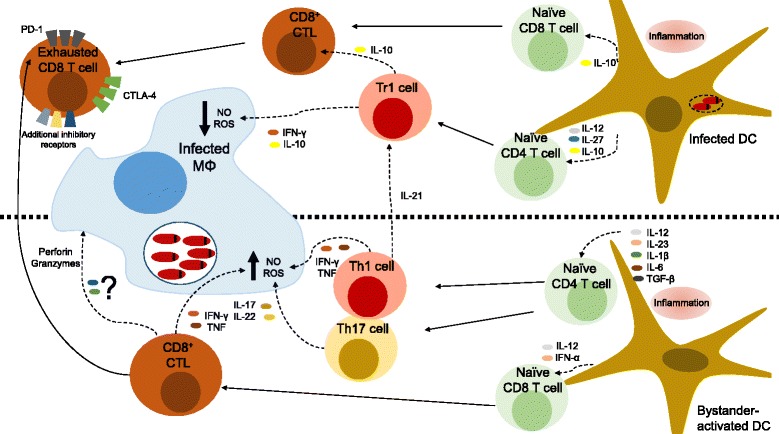
The immunologic environment in the spleen during visceral leishmaniasis. The picture aims to summarize the main host-protective responses occurring during VL in the spleen, as well as the major immune networks that promote parasite persistence (top half). Protective responses in the spleen are initiated by DCs exposed to parasite products, but not productively infected by *Leishmania* (bystander DCs). These secrete cytokines such as IL-12 or IL-23 that guide the differentiation of Th1 or Th17 cells, respectively, which, in turn, will produce IFNγ, TNF or IL-17 that maximize the capacity of infected macrophages to produce NO and ROS. In parallel, naïve CD8 T cells are primed by DCs in the presence of IL-12 and type I IFNs and differentiate into effector cells that further contribute to the protective response by producing IFNγ and TNF. Effector CD8 T cells may also degranulate perforin and granzymes and kill infected cells, although it remains unclear whether cytotoxic mediators play any protective role during VL. In contrast, in infected DCs the parasite hijacks the capacity of the cell to initiate protective responses (the mechanisms employed by *Leishmania* to subvert signaling pathways and impair host cell function fall outside the scope of this review and the reader is referred to recent reviews [167, 168]). The combined secretion of cytokines such as IL-12, IL-27 and IL10 by infected DCs leads to the differentiation of Tr1 cells that simultaneously produce IFN-γ and IL-10 and decrease the leishmanicidal capacity of the macrophage. In parallel, parasite persistence and possibly suppressive cytokines lead to the exhaustion of specific CD8 T cells, by upregulating the expression of inhibitory receptors such as PD-1, LAG-3 or additional unidentified receptors. These cells perform very limited effector function hence decreasing the capacity of the host to fight the parasite

A few hours after mice infection, mature DCs appear in T cell areas at the periarteriolar lymphoid sheaths (PALS) and produce IL-12 to initiate protective T cell responses [48, 50, 51]. Interestingly, priming DCs do not contain viable parasites, which led to the notion that protective T cell responses are induced by DCs activated in a bystander manner, hence resembling the early events in the liver mediated by bystander Kupffer cells (Fig. 1) [52, 53]. It is not clear how bystander DCs acquire parasite antigens for T cell priming. DCs may phagocytize parasite debris present in the splenic marginal zone or ingest macrophages containing digested parasites [13]. In mice infected with *L. donovani*, CD4 T cell activation can be detected in the first day after infection and the pool of parasite-specific splenic CD4 T cells increases several fold during the first weeks contributing to splenomegaly [20, 54].

In CD4 T cells, IL-12 signaling leads to nuclear translocation of Signal Transduction and Activator of Transcription-4 (STAT-4) resulting in induction of the transcription factor T-box transcription factor-21 (T-bet) and upregulation of IL-12 receptor (IL-12R) to prime for Th1 differentiation. T-bet, in turn, induces the cardinal Th1 cytokine IFN-γ that through autocrine signaling activates STAT-1 and further stabilizes the Th1 lineage [55]. There is substantial evidence indicating that all the components involved in Th1 differentiation are necessary for an effective response against visceral *Leishmania* [56–59].

Shortly after infection, splenic DCs are also capable of producing other members of the IL-12 family, including IL-23p19 [60], which may pair with IL12p40 to form biologically active IL-23 [61]. Along with additional cytokines present in the VL spleen, such as the Transforming Growth Factor-β (TGF-β), IL-6 or IL-1β; IL-23 promotes the differentiation of Th17 cells from naïve CD4 T cells [55]. Recent evidence suggests an important protective role for Th17 cells during VL (Fig. 1). Mice deficient for the IL17 receptor A (IL17RA) were more susceptible to *L. infantum* infection, exhibiting decreased numbers of splenic IFN-γ-producing CD4 T cells. Furthermore, IL-17A acts synergistically with IFN-γ to potentiate NO production in infected macrophages [62]. These studies in mice are supported by observations in human patients. For instance, analysis of cytokine responses in peripheral blood mononuclear cells (PBMCs) from symptomatic and asymptomatic VL patients revealed that the expression of Th17 cytokines was strongly associated with the asymptomatic state [63, 64]. Also, another study noticed negligible transcript levels of Th17-associated cytokines or transcription factors in splenic tissue from patients with active disease [65]. Interestingly, IL-17A appears to play a host-detrimental role during infections by cutaneous *Leishmania* species [66]. During parasite development in the sand-fly vector, *Leishmania* produces extracellular vesicles (also known as exosomes) which accumulate in the midgut and become part of the inoculum injected by the fly during feeding. By exacerbating the inflammatory response, particularly through the induction of IL-17A, exosomes cause larger lesion size and longer footpad swelling [67]. It would be interesting to explore whether exosomes described in visceral *Leishmania* species [68], similarly induce an exacerbation of the immune response in the skin or instead down regulate inflammation to allow a silent navigation to the viscera.

CD8 T cell-mediated immunity has been comparatively less studied than the CD4 T cell response during VL [69–71]. The expansion of splenic CD8 T cells after infection is impressive and may reach 10-fold within a 2 month period [20, 72]. CD8 T cells respond to IL-12 and type I IFNs by upregulating the T-bet and Eomesodermin (Eomes). These promote the expression of type I cytokines such as TNF or IFN-γ and cytotoxic molecules such as granzymes and perforin which allow CD8 T cells to perform effector function [73]. There is sustained evidence pointing to an important role of CD8 T cell-derived IFN-γ in the activation of infected macrophages to control parasite growth [70, 71, 74]. Less clear is whether CD8-mediated cytotoxicity plays any protective role (Fig. 1). Some studies in mice demonstrate that parasite-specific cytotoxic T lymphocytes (CTLs) generated during infection are capable of killing infected cells ex vivo [70, 74, 75], *via* mechanisms relying on the perforin/granzyme and Fas/FasL pathways [70]. However, it is not clear whether the parasite is killed concomitantly with the demise of the host cell. In vitro experiments indicate that parasites remain viable after CD8-mediated host cell lysis, but are eventually killed after infecting neighboring macrophages pre-activated with CD8-derived IFN-γ [76]. These observations suggest that the cytotoxic and cytokine-secreting functions of CTLs collaborate for efficient parasite killing, but such mechanism has so far not been demonstrated *in vivo*. The role of CD8 T cell effector function has been studied with more detail in infections with cutaneous *Leishmania* species [77]. In mouse models of CL and human CL patients, the current paradigm indicates that CD8 T cells producing IFN-γ contribute to the protective response against the parasite while CD8-mediated cytotoxicity leads to tissue pathology and promotes skin ulceration [78–82]. Whether a similar dichotomy in the effector functions of CD8 T cells is operative in VL remains unknown and is a matter of great interest in future studies.

In mice, after the initial period of parasite elimination by splenic macrophages, the parasite population is maintained at a constant size throughout the following two weeks, suggesting that parasite replication is balanced by parasite killing [19]. While able to control infection, the immune response appears to be far from optimal, as it cannot eradicate the parasite. By the third week after infection, parasite load in the spleen starts to increase slowly, signaling the onset of the chronic phase. By this time, infection is starting to resolve in the liver [19]. In the following sections, we will review the main immune networks responsible for parasite persistence in the spleen during chronic VL.

### Chronic visceral leishmaniasis: regulatory cytokines

Mice deficient in the immunosuppressive cytokine IL-10, or in which IL-10 signaling is blocked, are highly resistant to *L. donovani* infection [83, 84]. IL-10 is found in elevated levels in the serum, splenic aspirates, lymph nodes and bone marrow of VL patients [65, 85, 86] and is produced after *Leishmania* antigen stimulation of whole blood cultures from patients with active disease, but significantly decreases after drug cure [87]. Neutralization of IL-10 augments IFN-γ production in whole blood assays and promotes amastigote clearance in cultured splenic cells from VL patients [87, 88]. Together, all these pieces of evidence led to the conclusion that IL-10 is the major mediator of the immunological defects observed in the spleen during chronic VL [89, 90].

IL-10 is a general suppressive cytokine with a range of anti-inflammatory effects in several immune lineages [91]. During VL, IL-10 deactivates the leishmanicidal mechanisms of the macrophage and down regulates the expression of co-stimulatory molecules and MHC expression [89, 92, 93]. It also decreases the production of IFN-γ in T cells [87, 94] and inhibits DC migration to T cell areas [95].

IL-10 can be produced by multiple immune lineages [91, 96]. During VL, several cell types have been identified as sources of IL-10, including CD4 and CD8 T cells, B cells, NK cells, macrophages and DCs [53, 85, 97–100]. A major topic of interest in the past decade has been the identification of the relevant sources of IL-10 during VL. The best available evidence points to conventional IFN-γ-secreting Th1 cells as the most relevant source of pathological IL-10 during chronic experimental and human VL (Fig. 1) [17, 53, 85, 101, 102]. In mice, splenic CD4 T cells producing both IFN-γ^+^ IL-10^+^(sometimes denoted as type I regulatory T cells, Tr1) can be detected already at two weeks after parasite infection and attain a plateau by one month, representing 2 to 5 % of the total splenic CD4 T cell pool [53, 101].

A matter of upmost interest is to decipher the mechanisms underlying the regulatory switch that results in the induction of the IFN-γ^+^ IL-10^+^double producer CD4 T cells. Recent studies are unveiling a cytokinic network that works to maintain the suppressive environment during chronic VL. One study, employing splenocytes from human VL patients evidenced a role for T cell-derived IL-21 and myeloid cell-derived IL-27 in the induction of IL-10 in CD4 T cells [65]. Moreover, recent data points to a crucial role for DCs in promoting the regulatory switch in CD4 T cells (Fig. 1) [53, 101]. Indeed, DC-derived IL-27 and IL-12 appear to be involved in the induction of IL-10 in CD4 T cells [53, 101]. Interestingly, the suppressive-promoting capacity is restricted to infected DCs [53] and DC depletion between the third and fourth week after infection reduces pathology and enhances resistance to infection [101]. Finally, IL-10 signaling may contribute to additional IL-10 and IL-27 secretion by the infected macrophage, to continuously fuel this suppressive loop [65, 103]. Expression of IL-10 by Th1 cells is a widespread phenomenon that ensures a tight control over excessive activation that may cause pathology [96, 104]. During infections with the apicomplexans *Plasmodium* or *Toxoplasma*, the emergence of IL-10^+^ IFN-γ^+^ CD4 T cells is required to limit excessive pathology [105, 106]. Even during VL some evidence suggests that IL-10 may be host protective; particularly, in regulating a detrimental inflammatory response in the liver. Indeed, the extensive hepatic necrosis accompanying *L. donovani* infection in *TNF-*deficient mice may result from a concomitant defect in IL-10 induction [13]. Likewise, the severe hepatic pathology that follows *L. donovani* infection in *IL27R*^−/−^ mice involves CD4 T cells and may result from curtailed IL-10 induction [107].

The suppressive role played by DCs during chronic VL is not limited to the induction of Tr1 cells. Indeed, early work evidenced the expansion of a CD11^low^ CD45RB^hi^ DC population during *L. donovani* infection in mice that dampened T cell responses and induced antigen-specific tolerance *in vivo* [108]. The transfer of these CD11c^low^ DCs to DC-depleted and infected mice was able to restore splenomegaly and parasite burden to levels present in non-depleted mice, *via* a mechanism that did not involve the induction of Tr1 cells [101]. Finally, a recent study demonstrated that the early inflammatory milieu during VL promotes the activation of IRF-5 in DCs, which leads to upregulation and stabilization of the transcription factor Hypoxia Inducible Factor-1α (HIF-1α). HIF-1α, in turn, promotes the secretion of IL-10 by DCs, while limiting IL-12, which results in delayed expansion of specific CD8 T cells and their limited effector function, thus further supporting the suppressive role of DCs and IL-10 during VL (Fig. 1) [109].

Whilst much less studied than IL-10, TGF-β is another suppressive cytokine that has been linked with parasite persistence in VL [90, 110, 111]. Additionally, mice resistant to *L. infantum* infection become significantly more susceptible when injected with a viral vector expressing TGF-β [110].

### Chronic visceral leishmaniasis: T cell exhaustion

Chronic infections are characterized by a prominent impairment of T cell function, known as T cell exhaustion, which precludes an effective response in the long term [112]. Exhaustion proceeds progressively, paralleling the increase in pathogen burden [112]. Some functions, such as cytotoxicity, IL-2 production or proliferation are lost initially. Severe exhaustion is characterized by an inability to produce TNF, IFN-γ or to degranulate [113]. Apoptotic deletion is usually the final fate of an exhausted T cell. Nevertheless, exhausted T cells are capable of long-term survival, if their specific antigen remains present [112]. Evidence has convincingly linked the occurrence of T cell exhaustion with progressive and sustained expression of inhibitory receptors on effector T cells [114]. These include programmed death-1 (PD-1), cytotoxic T lymphocyte antigen-4 (CTLA-4) or lymphocyte-activation gene-3 (LAG-3) [112, 115, 116]. These receptors act by inhibiting T cell activation, thus precluding optimal effector function. T cell exhaustion was initially noticed over a decade ago in models of chronic viral infections [117, 118], but recent work unveiled a similar paradigm during chronic protozoan infections [119].

In mice infected with *L. donovani*, splenic CD8 T cells exhibit signs of functional exhaustion by the third week after infection, and severe functional impairment is evident after the fourth week, with abrogated production of IFN-γ, TNF, IL-2 and granzyme B (Fig. 1) [120]. Exhaustion is paralleled by increased expression of PD-1 in CD8 T cells and its ligand PD-L1 in splenic DCs. Treatment with an antibody blocking the PD-1/PD-L1 interaction rescued the functionality of parasite-specific effector/memory CD8 T cells, resulting in lower splenic parasite burden [120]. Interestingly, the recovery of CD8 T cell effector function after α-PD-1 treatment was only partial [120], suggesting that additional inhibitory receptors may contribute to the functional attrition of CD8 T cells during VL. In agreement, mice treated with a CTLA-4 blocking mAb 1 day after infection demonstrate significantly lower parasite burden by 1 month post-infection, consistent with the timing of CD8 T cell exhaustion [121, 122]. The relevance of these findings in mice has been confirmed in human VL patients, whose splenic CD8 T cells similarly exhibited functional impairment and augmented expression of PD-1 and CTLA-4 [123]. Contrasting with the wealth of evidence demonstrating CD8 T cell exhaustion, in CD4 T cells the phenomenon has been far less studied and is less understood [112]. In chronic canine VL, splenic CD4 T cell exhaustion is less severe than CD8 exhaustion and appears only in aggravated clinical stages of the disease [124].

Suppressive cytokines, such as IL-10 and TGF-β, have been consistently linked with T cell exhaustion in viral infections and cancer [125]. For instance, TGF-β directly enhances PD-1 expression in CD8 T cells [126]. Due to the elevated levels of these cytokines in the VL spleen it would be interesting to explore how these cytokines influence T cell exhaustion during VL and whether their blockade leads to an amelioration of the functionality of effector T cells (Fig. 1).

### Chronic visceral leishmaniasis: loss of splenic lymphoid architecture

The most striking clinical feature of both human and experimental VL is the impressive splenomegaly [30]. Concomitant with increased organ mass and size, a number of changes in the splenic microarchitecture occur [20]. These include disorganization of the white pulp, hypertrophy of the red pulp and disruption of the marginal zone. In the white pulp, germinal centers (GCs) disappear, and the PALS collapses [127]. Neovascularization is also prominent in both red and white pulp [128, 129].

In mice, the structural changes in the spleen start as the infection enters the chronic phase (around 3 to 4 weeks post-inoculation) [30]. Disorganization of the PALS is mediated by TNF and results mainly from the loss of gp38^+^ stromal cells. These are crucial for establishment and maintenance of the PALS by producing the chemokines Chemokine (C-C motif) Ligand-19 (CCL19) and CCL21, which attract naïve and memory T cells [95, 130, 131]. Concomitant with the disassembly of the PALS, an extensive remodeling of the splenic marginal zone also occurs, characterized by depletion of the MZM population [132]. Again, TNF appears to mediate the loss of MZMs, through a mechanism that is not clearly elucidated but may involve a direct apoptotic effect [132]. It is intriguing to note that TNF, the crucial cytokine responsible for the maturation of protective granulomas in the liver, is similarly the major factor responsible for the histopathological sequelae of chronic infection in the spleen [13].

A final significant alteration in the lymphoid architecture of the spleen during chronic VL is the loss of follicular dendritic cells (FDCs), leading to loss of GCs and B cell follicles, which become occupied by parasitized macrophages and plasma cells [13]. The structural changes that lead to loss of splenic lymphoid architecture during chronic VL disrupt cell-cell interactions that are crucial for effective immune responses, thus contributing to the suboptimal responses during chronic VL. For instance, the deletion of FDCs and concomitant disorganization of GCs impedes the long-term interactions between B cells and T follicular helper cells (Tfh cells) that are necessary for the production of specific antibodies capable of neutralizing the parasite [133].

### Antibodies, B cells and T follicular helper cells in visceral leishmaniasis

Experimental work performed over the past decades led to a prevailing view that considers B cells and antibodies of minimal importance for the protective immunity during VL. Indeed, hypergammaglobulinemia has long been recognized as one of the cardinal signs of VL, correlates positively with disease severity and decreases upon drug cure [134–138]. Early studies also demonstrated that most of the circulating IgGs are not parasite-specific, but instead result from polyclonal B cell activation [139, 140]. Indeed, autoantibodies are a recurrent finding in VL patients [141–143], frequently associated with proliferative glomerulonephritis [144].

**Fig. 2:**
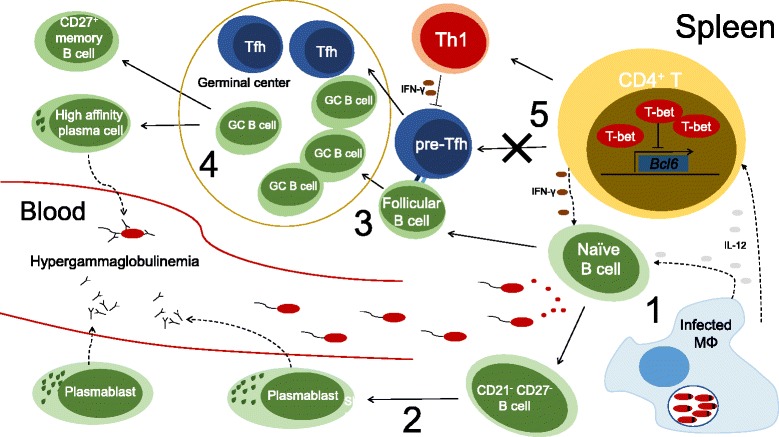
Dysfunctional humoral response during visceral leishmaniasis. The picture summarizes the sequence of events that lead to a suboptimal humoral response during visceral leishmaniasis, based primarily on data from our recent study in non-human primates compounded with evidence from additional studies. (1) Shortly after parasite inoculation, B cells are activated in a non-specific manner by soluble parasite products that act as B cell mitogens as well as by inflammatory mediators generated during the response to infection. (2) As a result, B cells with the atypical CD21^−^ CD27^−^ phenotype expand and eventually give rise to plasmablasts that produce copious amounts of immunoglobulin leading to the occurrence of hypergammaglobulinemia. (3) Some B cells appear to be activated in a specific manner *via* their BCR and follow the follicular pathway where they engage in cognate interactions with pre-Tfh cells. (4) If these interactions are productive, both cell types proceed to form a germinal center where Tfh cells promote affinity maturation of B cell for their specific antigen and direct the selection of the B cells clones with the highest affinity. B cells then exit the germinal center as high-affinity CD27+ memory B cells and plasma cells that produce antibodies with high affinity for the parasite. (5) However, the germinal center response is not sustained during the chronic phase of infection accompanying the decreasing numbers of Tfh cells. A strong Th1-polarizing environment is established in the spleen during VL, with high levels of expression of T-bet in CD4 T cells. Given that T-bet and the Tfh master transcription factor, Bcl-6, mutually repress each other’s expression, it is reasonable to speculate that the inflammatory environment during VL is unfavourable for the sustained differentiation of Tfh cells

In our recent study, employing a non-human primate model of VL, hypergammaglobulinemia was established early after infection and persisted during the chronic phase. Yet, the production of *Leishmania-*specific IgG was short-lived and decreased at chronic infection, implying that most antibodies produced are not specific for the parasite [17]. Analyzing the splenic B cell population we observed the expansion of memory B cells expressing CD27 after infection that contracted at the chronic phase, hence closely following the production of specific antibodies. We further observed the persistent expansion of a splenic B cell population with the atypical CD21^−^CD27^−^ phenotype that appeared responsible for the non-specific hypergammaglobulinemia (Fig. 2) [17]. These observations incited us to explore the dynamics of T follicular helper cells (Tfh cells) in the spleen of rhesus macaques infected with *L. infantum*. Tfh cells are a CD4 T cell helper subset specialized in coordinating GC reactions and providing crucial help to B cells in the production of high affinity antibodies [145]. Indeed, we observed the expansion of a splenic Tfh population in the first few weeks following parasite inoculation. Tissue imaging further evidenced that Tfh cells were able to infiltrate B cell follicles and GC during the acute phase. However, Tfh cells were mostly absent from the spleen at the chronic phase, hence paralleling the decline in CD27^+^ memory B cells and specific IgG [17]. Thus, our study in non-human primates suggests that the inability to maintain a sustained Tfh response during the chronic phase of infection may underlie the defects in the humoral response during VL (Fig. 2). Thus, it will be important to decipher the immune mechanisms behind this failure to maintain Tfh cells. One possibility may relate to the existence of a strong Th1-polarizing environment in the spleen during VL, with induction of expression of T-bet in CD4 T cells that directly represses the expression of the Tfh master transcription factor B cell lymphoma-6 (Bcl-6) (Fig. 2) [146]. Furthermore, the destruction of FDC networks and loss of GCs that occurs during VL, may also preclude a sustained Tfh differentiation and preclude their effector function.

During chronic VL, amastigotes are exposed to antibodies when they egress from heavily infected macrophages to infect new ones. The view that antibodies are detrimental to the host is supported by the observation that, when ingested by macrophages, IgG-opsonized amastigotes promote IL-10 secretion and inhibit IL-12 production [135, 147, 148]. However, ligation of FcγRs on the surface of macrophages and DCs may lead to pro- or anti-inflammatory outcomes, depending on the identity of the Fc receptors activated, IgG subclass or cell type [149, 150]. For instance, one study demonstrated that parasite-specific IgG is required for efficient *L. major* uptake and IL-12 production by DCs, suggesting that FcγR-mediated uptake has contrasting outcomes in DCs and macrophages [151]. Also, in a model of co-infection of *L. amazonensis* and *L. major* in mice, it was shown that resolution of the lesion required specific antibody that was able to enhance the microbicidal mechanisms in the macrophage by promoting ROS production [152, 153]. As such, the role of antibodies and Fc receptors during *Leishmania* infections is more complex than previously appreciated, with the outcome of FcγR ligation being clearly context-dependent.

Furthermore, the role of antibodies as regulators of the inflammatory response is not necessarily detrimental to the host. While B cell-deficient mice resolve *L. donovani* infection more rapidly than WT mice, such increased resistance comes at the cost of hepatic pathology. However, administration of immune serum to infected B cell-deficient mice alleviates pathology without decreasing the efficiency of hepatic parasite clearance, suggesting a tissue-protective role for antibodies [154].

Even conceding that parasite opsonisation by specific IgG is deleterious for the host, there are alternative mechanisms through which antibodies may contribute to the protective response against *Leishmania*; for instance by neutralizing parasite virulence factors. An illustrative example comes from the intracellular bacteria *Listeria monocytogenes.* A monoclonal antibody against listeriolysin, the pore-forming toxin of *L. monocytogenes*, was capable of blocking bacterial replication inside macrophages and provided resistance to infection in mice [155, 156]. Antibodies against *Leishmania* virulence factors, such as the metalloprotease gp63, have been detected in the sera of VL patients [157], but it is not clear whether they are capable of neutralization or play any protective function. As such, it is imperative to identify antibodies with neutralizing capacity and to evaluate whether their administration is capable of modifying the course of the disease to the benefit of the host.

Rather than considering the role of antibodies solely as pathological or irrelevant, it is perhaps wiser to acknowledge that these molecules may play both protective and non-protective roles during VL.

## Conclusions

The fight against the Neglected Tropical Diseases has received worldwide attention after the recent attribution of the 2015 Nobel Prize in Physiology or Medicine to William Campbell and Satoshi Ōmura for their development of a novel therapy against infections caused by roundworm parasites. Given its deadly and poverty-promoting features, control of visceral leishmaniasis should be given a high priority by policy makers of public health worldwide [158]. In 2007, the World Health Assembly delineated a proposal to drastically reduce the burden associated with the leishmaniases in the following years. By 2020, it is expected to identify and treat all cases of VL in the endemic regions of Africa, Europe and Americas and to reduce the prevalence below 1/10,000 in the endemic districts of the Indian sub-continent [159]. Such ambitious plan requires effective and affordable drugs. Unfortunately, all the available anti-*Leishmania* medicines suffer from more or less severe side-effects. In this context, immunotherapeutic approaches may help to restore immune function, potentially decreasing the dose of drug administered, while maintaining drug efficacy. Based on a detailed understanding of the immune events occurring during VL, one may envision multiple points at which immunotherapeutics may intersect the infection and improve the immune response to the parasite. Some immunotherapeutic strategies have already been employed in experimental models as well as human patients with variable degrees of success. Early work consisted in the administration of type I cytokines, such as IFN-γ and Il-12 [160, 161], which carries the risk of inducing excessive immunopathology. As an alternative, blocking the action of immune-suppressive factors should allow restoration of immune function in a more controlled manner. IL-10 blockade, in particular, has obtained remarkable success in lowering parasite loads, when combined with conventional treatment, in multiple studies in mice and splenocytes from human patients [162].

In the past few years, and following the advances in our understanding of the fundamental immunology of VL, new immunotherapeutic approaches have been proposed. As discussed above, blocking inhibitory receptors with the aim of reverting T cell exhaustion has obtained limited success, possibly because we still do not completely understand the factors governing T cell exhaustion during VL. Nevertheless, reverting T cell exhaustion has enjoyed a remarkable success in the treatment of viral infections and cancer [125]. As such, efforts should continue to elucidate the role of additional inhibitory receptors and suppressive cytokines in T cell exhaustion during VL. The remodelling of lymphoid structures in the spleen during VL represents an additional target for immunotherapeutics, as restoration of normal lymphoid architecture may potentially improve immune function. Indeed, a study in mice demonstrated that the administration of an anti-angiogenic drug prevents splenic vascular remodelling and loss of lymphoid architecture during VL. As a result, the numbers of IFN-γ-producing CD4 T cells augmented and the efficacy of antimonial therapy improved drastically [128]. Finally, as discussed before, administration of neutralizing or otherwise protective antibodies should not be discarded as a future potential immunotherapeutic approach for VL, although this strategy has not yet been tested by researchers, possibly because we still do not know whether such antibodies can be produced during VL. Ultimately, effective and long-lasting control of VL will depend on the development of a human vaccine. Unfortunately, despite the remarkable progress obtained in identifying new immunogenic parasite antigens and increasingly powerful adjuvants, the goal of controlling VL through vaccination remains a formidable challenge [163]. Such lack of success results, at least in part, from the incomplete knowledge on the memory T cell subsets that vaccination should induce in order to confer protection. In this respect, VL vaccinology may profit from recent advances made in cutaneous models of leishmaniasis. Previous work identified circulating subsets of effector (TEM) and central memory (TCM) T cells generated in mice that clear their primary cutaneous infections and that, when transferred to naïve hosts, could confer partial protection [164, 165]. More recently, a skin-resident CD4 T cell memory subset, similarly generated in mice who resolved their primary infections, was shown to rapidly produce IFN-γ at the site of secondary challenge and boost the recruitment circulating T cell memory subsets. The simultaneous transfer of both skin-resident and circulating memory T cells to naïve mice conferred complete protection to *L. major* infection [166]. Whether skin-resident memory T cells can be generated and confer protection during visceral *Leishmania* infection remains unknown. Nevertheless, a vaccine approach capable of constraining the visceralizing parasites to the skin holds much promise as it would block colonization of the viscera, where these species are perfectly adapted for survival and subversion of the immune response.

## Abbreviations

APCs: antigen-presenting cells
AIDS: Acquired Immunodeficiency Syndrome
Bcl-6: B cell lymphoma-6
BM: bone marrow
CCL19: chemokine (C-C motif) ligand-19
CTLA-4: cytotoxic T lymphocyte antigen-4
CTLs: cytotoxic T lymphocytes
CXCL10: chemokine (CXC motif) Ligand-10
DC: dendritic cell
Eomes: Eomesodermin
FDCs: follicular dendritic cells
GCs: germinal centers
HIF-1α: Hypoxia Inducible Factor-1α
IFN-γ: interferon-γ
IL-12: interleukin-12
IL-12R: IL-12 receptor
IL17RA: IL17 receptor A
iNKT: invariant natural killer T
IRF: Interferon Regulatory Factor
LAG-3: lymphocyte activation gene-3
LPG: lipophosphoglycan
MMM: marginal metallophilic macrophages
MZM: marginal zone macrophages
NO: nitric oxide
PALS: periarteriolar lymphoid sheaths
PBMCs: peripheral blood mononuclear cells
PD-1: programmed death-1
ROS: reactive oxygen species
STAT-4: Signal Transducers and Activators of Transcription-4
T-bet: T box transcription factor-21
TCM: central memory T cells
TCR: T cell receptor
TEM: effector memory T cells
Tfh: T follicular helper cells
TGF-β: Transforming Growth Factor- β
Th1: T helper 1
TNF: tumor necrosis factor
Tr1: type I regulatory cells
VL: visceral leishmaniasis
